# Targeting AXL cellular networks in kidney fibrosis

**DOI:** 10.3389/fimmu.2024.1446672

**Published:** 2024-11-04

**Authors:** Sturla M. Grøndal, Magnus Blø, Linn I. H. Nilsson, Austin J. Rayford, Akil Jackson, Gro Gausdal, James B. Lorens

**Affiliations:** ^1^ Department of Biomedicine, University of Bergen, Bergen, Norway; ^2^ BerGenBio ASA, Bergen, Norway; ^3^ Clinical Development, BerGenBio Ltd., Oxford, United Kingdom

**Keywords:** Axl, bemcentinib, UUO (unilateral ureteral obstruction), mass cytometry (CyTOF), fibrosis, inflammation

## Abstract

**Introduction:**

The incidence of chronic kidney disease (CKD) is increasing, in parallel with risk factors including obesity and diabetes mellitus. AXL plays a central role in CKD, providing a rationale to evaluate clinical AXL targeting agents.

**Methods:**

To determine the efficacy and underlying molecular mechanisms of AXL inhibition in CKD, we employed a murine unilateral ureteral obstruction (UUO) model preventively treated with a selective AXL kinase inhibitor (bemcentinib) during disease progression. We isolated kidneys at an early (3 days) or late (15 days) timepoint and profiled the cell populations using mass cytometry.

**Results:**

Preventive treatment with bemcentinib significantly attenuated fibrosis in the UUO model. The anti-fibrotic effect correlated with a decrease in mesangial cells and inhibition of innate immune cell infiltration, while the proportion of epithelial cells increased. We mapped AXL expression to a unique network of cells in the kidney: mesangial cells, pericytes, macrophages and dendritic cells.

**Discussion:**

We propose that AXL targeting affects an important cellular interaction network underlying fibrotic progression. These results support the clinical application of AXL targeting agents to treat CKD.

## Introduction

Chronic kidney disease (CKD) manifests as a reduced capacity of the kidneys to filter blood leading to increased concentrations of waste products, such as creatinine and urea. CKD does not present immediate symptoms, which allows the disease to progress unnoticed. It is estimated that in 2017 approximately 11.1% of the global population suffered from CKD stages 1-5, representing nearly 850 million people (1). The prevalence of CKD is increasing, mostly due to the increased incidence of diabetes and obesity. In 2017, CKD was the 12th leading cause of death globally (2), and is estimated to rise to fifth place by 2040 (3). From 1990 to 2017, CKD demonstrated an increase of 41.5% in all-age mortality (2).

CKD treatment comprises anti-hypertensive and antidiabetic therapy, and symptomatic treatment including erythropoietin, diuretics and statins. CKD progression to end-stage kidney disease requires dialysis and transplantation. SGLT2 inhibitors were recently shown to delay progression to end-stage kidney disease by 30%, demonstrating that drugs may be a feasible treatment option (4–6).

The AXL receptor tyrosine kinase (RTK) is activated by its protein ligand Gas6. AXL-Gas6 complexes bind exposed phosphatidylserine (PS) on membranes of apoptotic bodies, viruses and stressed cells (7). AXL is expressed by endothelial and myeloid cells and is important for phagocytosis and is upregulated by inflammation and hypoxia in several cell types (8). AXL is expressed in mesangial cells and is reported to drive their proliferation (9, 10). AXL expression is associated with kidney inflammation in humans (11), while, AXL inhibition has been shown to ameliorate kidney inflammation and fibrosis in several mouse model studies (12–14). During inflammation, the extracellular domain of AXL can be cleaved, generating soluble AXL (sAXL) in the blood. Elevated serum sAXL levels has been observed in CKD patients, suggesting its potential use as a kidney disease biomarker (15, 16).

Although AXL inhibition reduces kidney fibrosis, the underlying molecular mechanism is poorly understood (12, 14). We therefore applied single-cell mass cytometry profiling to measure cellular changes during UUO-induced kidney fibrosis and identify AXL-expressing cellular networks that govern fibrotic progression.

## Methods

### Animal model and experimental design

Male C57Bl/6 mice aged ≥ 6 weeks from Janvier were kept at the laboratory animal facility at the Department of Biomedicine at the University of Bergen in Norway. The experiments were conducted according to guidelines and with the approval of the Norwegian Food Safety Authority (FOTS). Water and chow were provided ad lib.

Unilateral ureteral obstruction was performed as described in (12). Mice were anesthetized with isoflurane and kept on a heating pad during surgery. Skin surrounding the area of the left kidney was shaved, washed, and disinfected with 2% iodine solution. A subcoastal incision was made to access the left kidney. The ureter was obstructed with a silk ligature at the lower pole of the kidney. Peritoneum and muscle incision were closed with absorbable suture and skin clamps. The mice were treated with subcutaneous analgesia (Buprenorphine 0.1 mg/kg) directly after surgery and monitored hourly for the first 6 hours thereafter.

### Drug treatment

Bemcentinib-treated animals were dosed with 50 mg bemcentinib per kg mouse weight, solubilized in vehicle (0.5% (w/v) hydroxypropyl methylcellulose, 0.1% (w/v) Tween 80) at 5 mg drug per ml vehicle, yielding a final dose volume of 10 ml per kg mouse weight. The drugging was performed, twice daily, 8 hours apart, through oral gavage starting from the day prior surgery. On the day of surgery, mice were only drugged once before surgery with a double dose at 100 mg/kg. The last day of drug treatment was the day of euthanasia.

### Tissue collection and processing

Mice were subjected to general anesthesia using Sevoflurane, followed by heart puncture and finally euthanasia by cervical dislocation. Both kidneys were collected.

One-fourth of each kidney was snap-frozen in liquid nitrogen while another fourth was formalin-fixed and paraffin embedded (FFPE), and remaining half was cryopreserved in FBS (10% DMSO) at -80°C for later dissociation into a single cell suspension.

### Sirius red staining

Sirius red staining and analysis was performed as described in (12). In short, FFPE sections (3 µm) were deparaffinized in xylene, rehydrated in decreasing concentrations of ethanol, and incubated in 0.1% SR F3B (BDH Laboratory supplies, cat #34149) in saturated aqueous picric acid for 30 minutes.

Stained slides were scanned with brightfield imaging using the ScanScope system (Aperio, Vista, California, USA) at 0.23 µm pixel resolution. Digital slide images were viewed, annotated and analyzed in ImageScope 12 (Leica Biosystems, Nußloch, Germany). Sirius red quantification was performed using the Color Deconvolution algorithm version 9.1 (Aperio). Custom calibration of the 3-color stain deconvolution components (red, green, and blue optical densities, RGB ODs, for each stain “color”) was performed by selecting and annotating representative image regions containing pure areas of unstained tissue (Color 1), tissue with maximal (saturated) sirius red staining (Color 2), and no tissue (Color 3) and assigning the average RGB ODs from the representative areas as the deconvolution components for each stain color. The deconvolution parameters as well as optimized intensity thresholds for the deconvolved sirius red channel to categorize weak-, medium-, and strong-positive pixels (190, 170, and 140, respectively) were exported as a macro and applied to annotated whole-kidney regions in each slide. The sum of all positive pixel percentages (low + medium + high, “Percent Total Positive” column in the macro output data) was used to represent sirius red staining intensity in comparisons between samples.

### Immunofluorescence

Two or three FFPE sections (4 µm) per treatment group (15 days ligated vehicle, 15 days ligated bemcentinib, and sham) were deparaffinized in xylene, rehydrated in decreasing concentrations of ethanol, and incubated at 95°C for 30 min in Antigen retrieval solution pH 9 (S2367, DAKO). Slides were washed in MilliQ and blocked with PBS (5% BSA, 0.1% Tween 20) for 30 min. Sections were encircled with a hydrophobic barrier ImmEdge pen (H-4000, Vector Laboratories) and stained with AXL (cat# AF854, R&D Systems), F4/80 (cat# 70076S, Cell Signaling Technology), or alpha-smooth muscle actin (cat# 14-9760-82, ThermoFisher Scientific) overnight at 4°C. The next day the slides were washed four times in PBS (0.1% Tween 20) and stained with secondary antibodies against rabbit (A-21244, Thermo Fisher Scientific) or goat (A-21447, ThermoFisher Scientific) conjugated to Alexa Fluor 647 for one hour at room temperature. Subsequently, slides were washed three times with PBS (0.1% Tween 20) and incubated with DAPI at room temperature. Finally, slides were washed once in PBS (0.1% Tween 20) and twice in MilliQ and mounted with ProLong Diamond Antifade Mountant (P36965, ThermoFisher Scientific).

Sections stained with ASMA were imaged using Andor Dragonfly 505 confocal, while sections stained for AXL and F4/80 were imaged with Olympus VS120 S6 Slide scanner. Slides were imaged at the Molecular Imaging Center, Department of Biomedicine, University of Bergen.

Mean signal intensity was quantified using QuPath (v. 0.5.1) (17). Tissue of good quality was annotated. Areas with poor quality were not included in the analysis. For AXL, only cortex was annotated, while for ASMA and F4/80, both cortex and medulla were annotated. Mean signal intensity was calculated using *Compute intensity features* with default settings: Pixel size=2µm, Region=ROI, Basic features=Mean.

### Cell dissociation for mass cytometry

Kidney samples were thawed in a water bath (37°C, 1 min) until only a small ice crystal remained. The samples were centrifuged at 300g for 3 minutes and supernatant was discarded. 1.256 ml dissociation solution from Multi Tissue Dissociation Kit 1 (Miltenyi) was prepared by mixing 1.175 ml Buffer X with 50 µl Enzyme D, 25 µl Enzyme R, and 6.25 µl Enzyme A and added to each kidney sample. The samples were dissociated at 37°C for 30 minutes in a rotating wheel inside an incubator. The remaining fibrotic tissue pieces were further mechanically dissociated with two slow passes in a Dounce homogenizer. The homogenizer was washed with 500 µl RPMI 1640 containing 0.25 mg/ml DNase I (DN25, Sigma) to collect any remaining cells. To prepare for the next sample, the homogenizer was washed with Milli-Q water followed by 70% ethanol between samples to prevent cross contamination. Mechanical dissociation of all samples per batch was completed within 10 minutes. Samples were then placed back into the rotating wheel for another 30 minutes. Finally, samples were centrifuged at 300g for 3 minutes and supernatant was discarded.

Dissociated samples were resuspended in wash solution (DPBS with Mg/Ca, 6% bovine serum albumin, 0.25 mg/ml DNase I) supplemented with 0.01 µM rhodium intercalator (Fluidigm) and incubated for 10 minutes at room temperature for viability staining. During incubation, cells were passed through a 70 µm strainer. Samples were allowed to rest for another 10 minutes at 37°C before being centrifuged at 300 g for 3 minutes. Supernatant was aspirated and cells were first resuspended in 437.5 µl DPBS before fixation by the addition of 62.5 µl paraformaldehyde (0.2 µm filtered, 16%, EMS) for a final concentration of 2% for 10 minutes at RT. 1 ml 1M Tris (pH=8.7) was used to quench the fixation. Samples were then centrifuged at 300 g for 5 minutes and supernatant was discarded. Each sample was resuspended in DPBS (10% DMSO, 1% BSA, 0.25 mg/ml DNase I) and placed in -80°C.

60 samples were barcoded using a combination of 20-plex barcoding kits and monoisotopic cisplatin solutions (194, 195, 196, and 198) according to manufacturer instructions with some modifications. Up to 3 million cells per sample were washed in 500 µl barcode perm buffer (Fluidigm). 5 µl barcode solution was added to 95 µl barcode perm buffer containing 125 nM cisplatin and, immediately after mixing, transferred to the sample. Samples were incubated at RT in a rotating wheel at 20 rpm for 60 minutes. After incubation samples were topped off with barcode perm buffer and then washed once in 1ml DPBS (1% BSA, 0.25 mg/ml DNase I). Samples were then pooled using 200 µl DPBS (10% DMSO, 1% BSA, 0.25 mg/ml DNase I) per sample and frozen at -80°C.

### Antibody conjugation and staining

Antibody conjugation was performed using commercial kits according to manufacturer’s protocols. Conjugation of 157Gd labelled antibodies employed Ionpath’s protocol, while conjugations using lanthanides and cadmiums employed Fluidigm’s protocols. To conjugate indium and lanthanum, the salts were first dissolved in L-buffer (Maxpar^®^ X8 Antibody Labeling Kit, Fluidigm) to yield a stock solution of 1 000 mM. A diluted solution of 50 mM metal solution was used for antibody conjugation according to Fluidigm’s lanthanide protocol.

Pooled barcoded cells were washed (DPBS, 1% BSA, 0.25 mg/ml DNase I) and strained through a 40 µm strainer. The cells were centrifuged and supernatant aspirated. Cells were then blocked with Fc-binding antibodies and heparin (18) for 10 minutes at RT, followed by addition of surface antibody staining for 30 min RT. The cells were washed twice with wash solution and once with PBS. The cells were then fixed in 2% PFA for 1 minute RT before quenching with 2M Tris, centrifuging and discarding the supernatant. Cells were then permeabilized on ice with -20°C methanol for 10 minutes, after which they were washed, blocked with Fc-binding antibodies and heparin and stained intracellularly similar to above. The cells were washed twice in wash solution and once in PBS and finally fixed in PBS (2% PFA, 2 mM EDTA, 50 nM iridium intercalator) and left in the fridge overnight. The following day, 2M Tris was used to quench the fixation followed by washing. DMSO was added and five aliquots were cryopreserved. Before acquisition, cells were thawed, washed twice in Milli-Q and resuspended in 1x EQ-bead solution and acquired on the Helios mass cytometer. The Helios passed tuning requirements and cells were acquired without concurrent FCS-processing to reduce acquisition time.

### Mass cytometry analysis

After acquisition, the resulting IMD files were processed using a lower convolution threshold of 400, min event duration of 7, max event duration of 150, sigma of 3, noise reduction, and gaussian discrimination, and dual count start of 1.

FCS-processing was performed using R 4.1.3 and 4.3.3 (19) and R Studio (20). FCS-files were normalized using premessa (21). XML-gates of normalized files were exported from FlowJo 10.7.1. XML gating files were applied by the R-package flowUtils (22). Gated files were then concatenated before debarcoding with CATALYST (23). Debarcoded files were concatenated into a single data frame with a filename column. Events with <20 ion counts from markers used in the analysis were removed. This yielded 5 331 610 events for further analysis.

Spillover was compensated by CATALYST with a previously acquired spillover matrix. Compensated data was transformed using asinh with a cofactor of 5. UMAP was performed using the cosine metric with the RcppHNSW and uwot (24, 25), while clustering was performed using PARC (26) with the parameters knn=20, resolution_parameter=1.5, too_big_factor=0.7, n_iter_leiden=4, distance=l2, hnsw_param_ef_construction=500, and small_pop=300, through reticulate (27).

The count matrix, containing the counts of each cluster in every sample, was converted into an acomp object and centered log ratio (CLR)-transformed using the cdt function in the compositions package (28), which is designed to treat compositional data. A mislabeled sample, originally labeled as nonligated kidney, was removed as it demonstrated unequivocal signs of ligation. Partial least squares (PLS) modelling was performed on acomp transformed data using the mixOmics R package (29). LASSO was performed using the cv.glmnet with lambda decided from lambda.1se (30). Correlation between cluster size and treatment was performed using Kendall rank correlation coefficient and the acomp-transformed compositional cluster size.

To test for differences in clusters across treatment groups, a multivariate Cramer’s test (31) was applied using the R-package cramer (32). P value was determined by Monte-Carlo-bootstrapping with 100 000 replicates and adjusted using Benjamini-Hochberg procedure. T-test was used to compare clusters between two groups at a time. Benjamini-Hochberg was used to adjust for multiple hypothesis testing. P < 0.05 (*), P < 0.01 (**), P < 0.001 (***), P < 0.0001 (****).

## Results

We subjected male mice to UUO for 3 or 15 days to induce inflammation and kidney fibrosis. Animals were preventively treated with the small molecule AXL kinase inhibitor bemcentinib or vehicle control twice per day starting from the day prior surgery. One group of animals was subjected to a sham operation where mice only received the incision and no subsequent ligation. Kidneys were harvested at indicated timepoints, stained for collagen with sirius red, and dissociated for single-cell mass cytometry analysis.

### Mass cytometry captures cellular changes during kidney fibrosis

**Figure 1:**
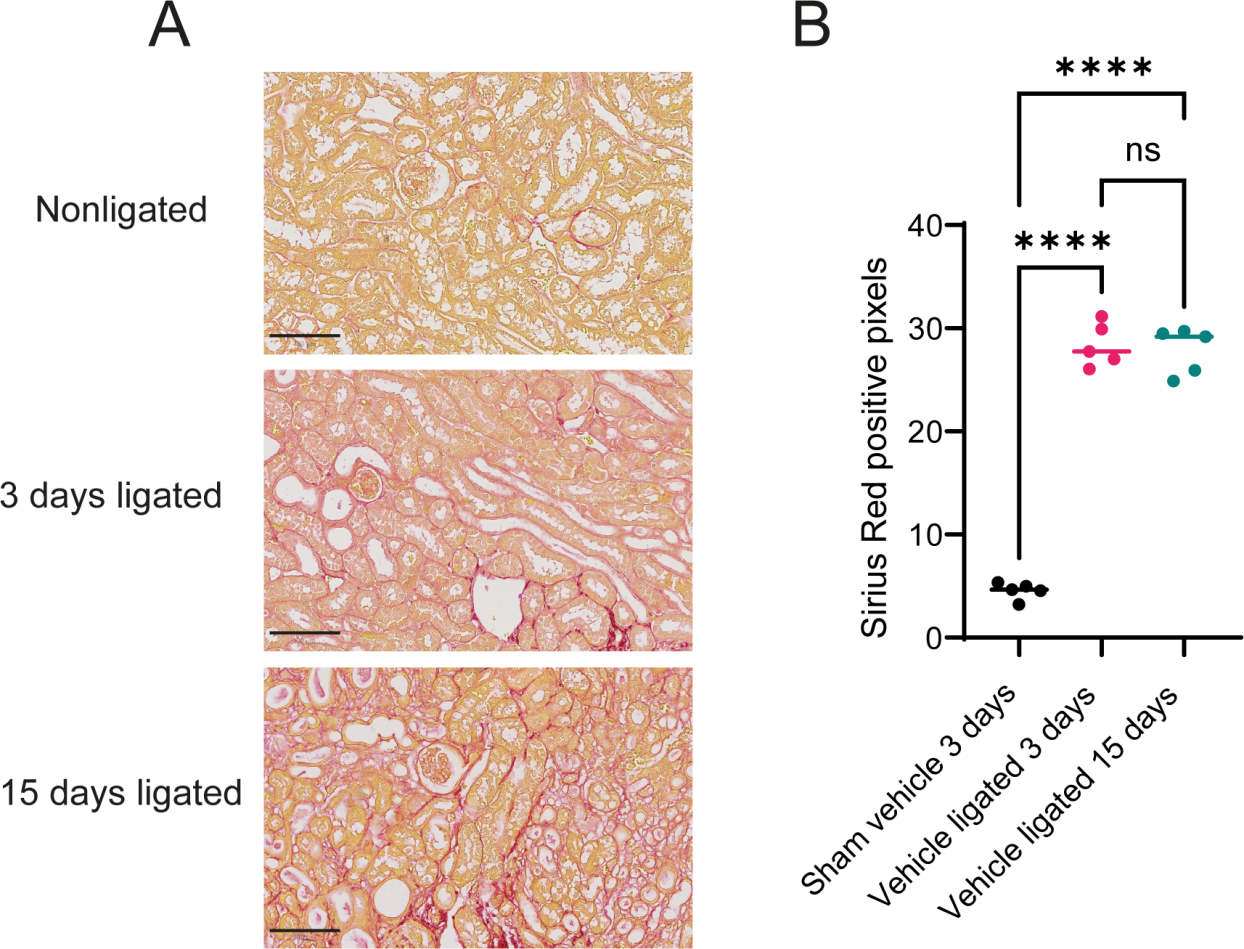
Unilateral ureteral obstruction increases sirius red staining **(A)** Sirius red staining of kidneys ligated for 0, 3, or 15 days from vehicle treated mice. Scale bar is 100 µm **(B)** Sirius red staining quantification. One-way ANOVA (P value < 0.0001) followed by *post-hoc* Tukey. ****P value < 0.0001; ns P value > 0.05.

To assess fibrosis development, we quantified sirius red staining (a marker for collagen deposition) in mouse kidney sections (**Figure 1A**). As expected, kidneys from sham mice treated with vehicle for 3 days demonstrated very low levels of sirius red staining. Kidneys subjected to 3 or 15 days of ligation, however, displayed significantly increased sirius red staining, indicative of onset of fibrosis, demonstrating the pro-fibrotic effectiveness of the UUO procedure (**Figure 1B**). Additionally, kidneys from sham operated mice or from mice subjected to UUO-ligation and treatment with bemcentinib or vehicle for 15 days were stained for AXL, alpha-smooth muscle actin (ASMA), and F4/80 (**Supplementary Figure 3**). ASMA and F4/80, markers of fibrosis and inflammation, respectively, demonstrated similar results with highest expression in kidneys ligated for 15 days treated with vehicle, reduced staining intensity in kidneys ligated for 15 days treated with bemcentinib, and the lowest expression in sham kidneys. Cortical expression of AXL was found to be similar in kidneys from mice subjected to UUO-ligation 15 days regardless of bemcentinib treatment, while kidneys from sham mice had a somewhat reduced staining.

**Figure 2:**
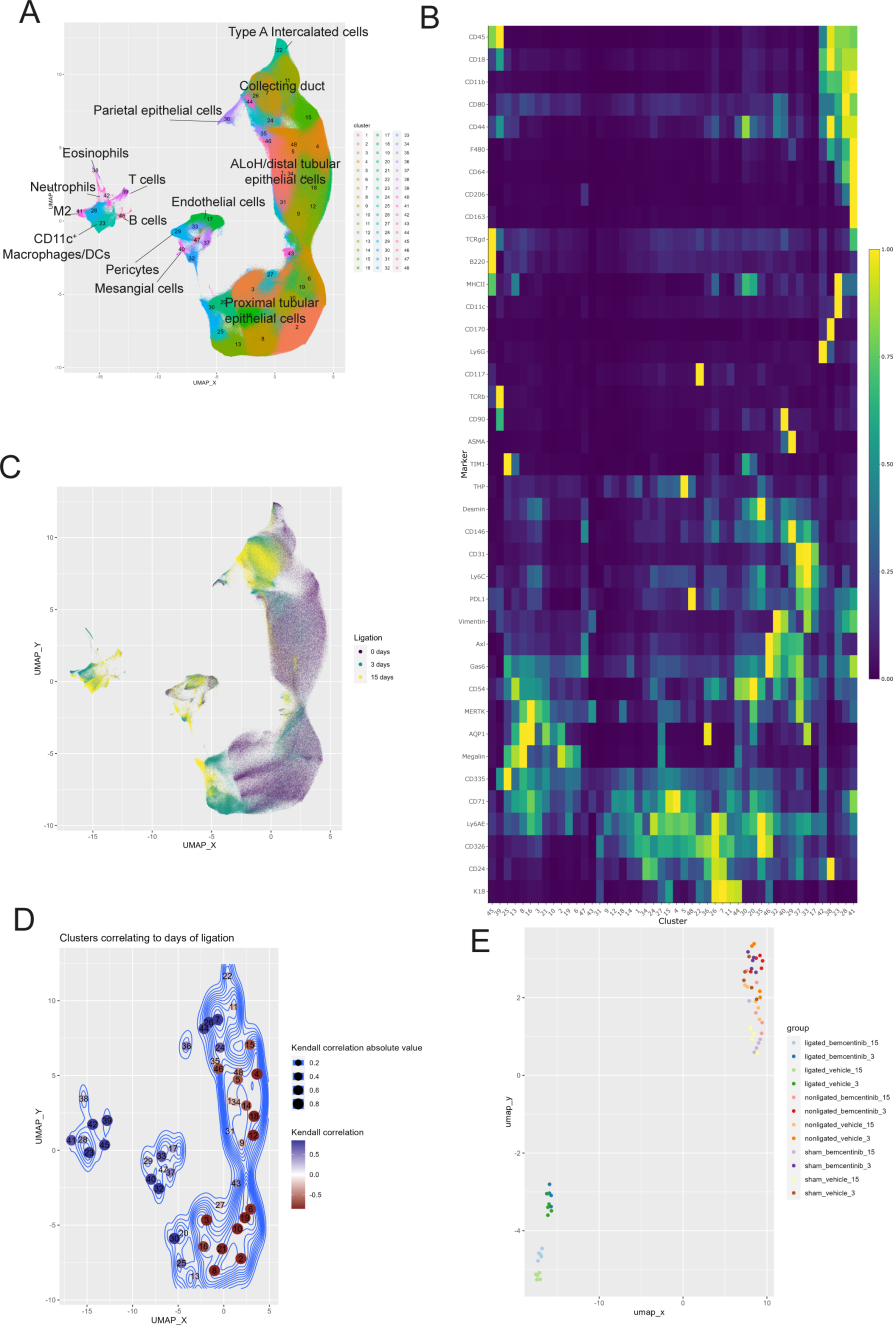
Temporal dynamics of kidney cell types after UUO **(A)** UMAP of kidney cells colored by cluster membership. ALoH, Ascending limb of loop of Henle; M2, M2 polarized macrophages **(B)** Heatmap displaying scaled mean expression of indicated markers in every cluster. **(C)** UMAP showing kidney cells from vehicle treated mice colored by ligation duration: purple for 0 days, green for 3 days, and yellow for 15 days. The same number of cells are used to plot each condition. **(D)** UMAP with clusters colored by Kendall’s correlation coefficient between transformed cluster size with fibrosis development. **(E)** UMAP of all kidney samples based on transformed abundance of the identified cell types. Sham and nonligated samples group in the upper right, while ligated kidneys gather in the lower left.

Using the clustering algorithm PARC (26) we identified 48 distinct clusters in the mass cytometry data set (**Figure 2A**, **Supplementary Table 1**). The clusters were phenotyped based on marker expression and included a diverse set of cell types including macrophages, mesangial cells, proximal tubular cells, endothelial cells, and epithelial cells from the ascending limb of loop of Henle (ALoH) and collecting duct (**Figure 2B**). By comparing cells from the kidneys of vehicle-treated mice subjected to 0, 3, or 15 days of ligation based on similarity of marker expression levels we found that the single-cell landscape profiled by our mass cytometry approach was able to capture changes occurring during development of fibrosis (**Figure 2C**). The cells from the different treatment groups did not overlap, indicating that the markers used in our panel adequately captured the cellular changes occurring during fibrosis development.

To see which cell type clusters changed with fibrosis development we correlated CLR-transformed cluster abundance with time (sham, 3 days ligation, 15 days ligation) (**Figure 2D**). This allowed us to study how the different cell types respond to fibrosis development. Our results demonstrated that the CLR-transformed abundance of all types of immune cells increased during development of fibrosis. Further, stromal cells, like mesangial cells, pericytes, and endothelial cells also increased, while numerous clusters of epithelial cells encompassing proximal tubules, and ALoH/distal tubules were reduced.

To visualize overall differences in cell type composition between treatment groups and timepoints, we plotted samples based on the UMAP-derived similarity of cluster abundances in each sample (**Figure 2E**). The UMAP plot demonstrated that sham and non-ligated kidneys (upper right corner) were highly similar, while kidneys 3 days and 15 days post-ligation (lower left corner) were similar to each other and clearly distinct from sham and non-ligated kidneys.

**Figure 3:**
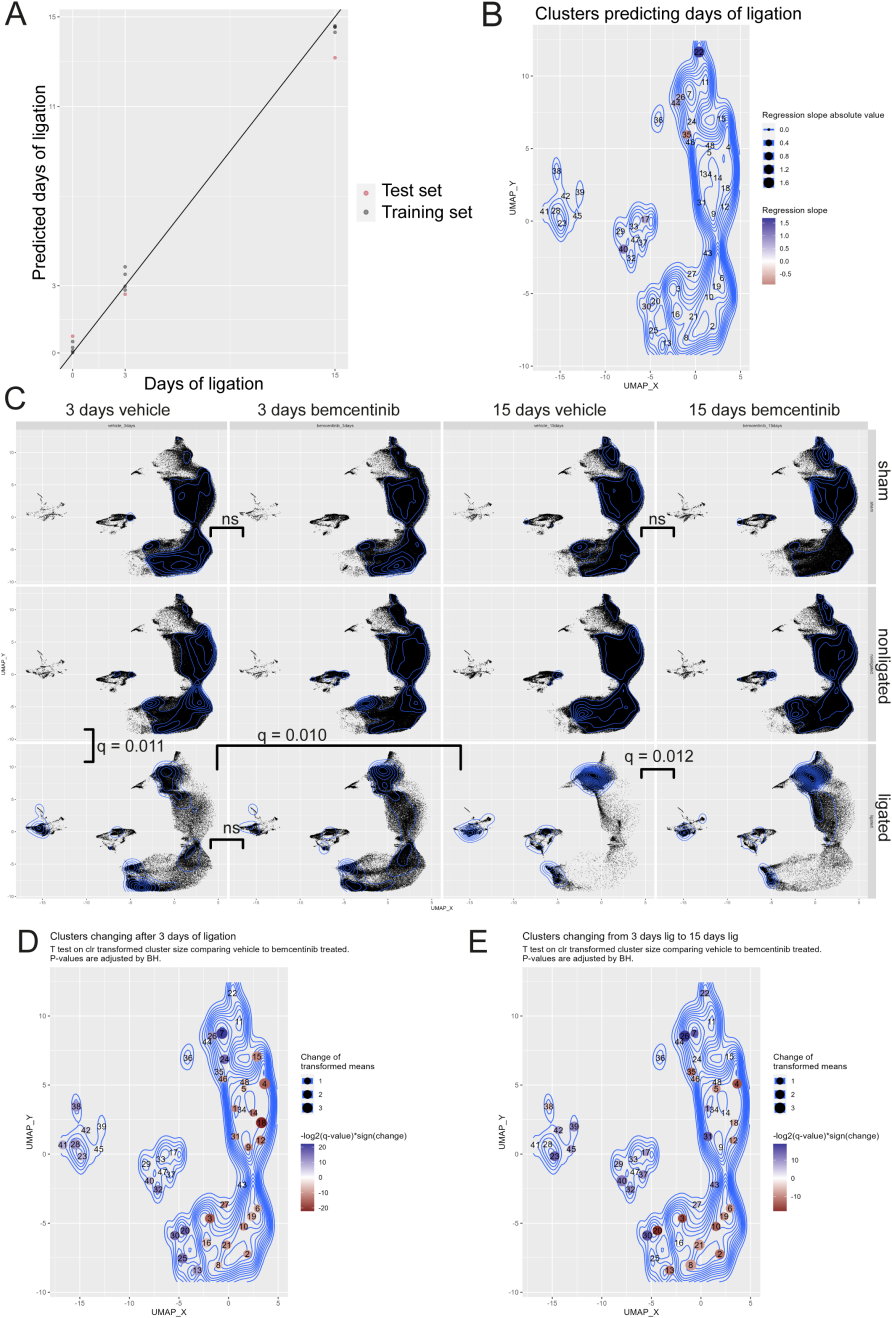
LASSO modelling and multivariate testing **(A)** LASSO model estimates of days of ligation (Y-axis) vs actual days of ligation (X-axis) based on transformed abundance of the cell types. N=4 replicates were used per group for training, and n=1 was used to test the model. **(B)** UMAP of clusters colored by LASSO slope. **(C)** UMAP of kidneys studied with mass cytometry with each facet displaying a treatment group. Topographical lines indicate density. Each plot contains the same number of cells. Multiparametric Cramer’s test was used to test for differences between vehicle and bemcentinib (green arrows) or nonligated, 3 days ligated, and 15 days ligated (blue arrows). P-values were adjusted for multiple hypothesis testing with Benjamini-Hochberg. **(D)** UMAP with clusters colored according to significant change in transformed abundance between nonligated 3 days vehicle vs ligated 3 days vehicle. Blue clusters increase, while red clusters decrease, after 3 days of ligation. Nonsignificant changes (adjusted P value > 0.05) are white. **(E)** UMAP with clusters colored according to significant change in transformed abundance between ligated 3 days vehicle vs 15 days ligated. Blue clusters increase, while red clusters decrease, from 3 to 15 days of ligation. Nonsignificant changes (adjusted P value > 0.05) are white. P values were calculated using multivariate Cramer’s test in **(C)** and Wilcoxon rank-sum in **(D, E)**. P values were adjusted for multiple hypothesis testing using Benjamini-Hochberg.

Given that we were able to describe how cell types change during fibrosis development, we reasoned that it would be possible to predict the number of days post-ligation, i.e. fibrosis development, from the abundance of the clusters. To highlight the most predictive clusters, we made a least absolute shrinkage and selection operator (LASSO) model to estimate the number of days a kidney had been ligated based on the CLR-transformed abundance of each cell type. To test the LASSO model, we estimated the number of days post-ligation for vehicle-treated kidneys that had not been used for training the model (**Figure 3A**). These test samples were closely grouped with the trained samples and the predicted number of days post-ligation closely resembled the true number. By studying the model, we found that CD117^+^ Type A intercalated cells (cluster 22) and CD90^+^ mesangial cells (cluster 40) were the clusters with the highest regression slope (**Figure 3B**). CD24^+^CD54^+^K18^+^ collecting duct epithelial cells (clusters 26 and 44) also had a positive, but substantially lower, slope.

To assess whether the treatments resulted in any statistically significant changes, we performed a multivariate nonparametric Cramer’s test on the CLR-transformed abundance of each cluster across the sample groups (**Figure 3C**). We found that at 3 days post-ligation, non-ligated kidneys from vehicle-treated animals were significantly different from corresponding ligated kidneys. We also found that kidneys ligated for 3 days were significantly different from kidneys ligated for 15 days.

### Temporal cell type-specific changes

Upon further inspection into the differences between non-ligated kidneys and kidneys ligated for 3 days, we found that ligation led to a significant increase in the CLR-transformed abundance of immune cells, mesangial cells and CD24^+^CD54^+^K18^+^ collecting duct cells and CD54^+^Tim1^+^ proximal tubular epithelial cells (**Figure 3D**). Further, ligation induces a reduction in normal epithelial cells from proximal tubules to ALoH/distal tubules. In terms of relative abundance, immune cells (clusters 23, 28, 38, 39, 41, 42, 45) increased from 0.7% to 11.8%, CD90^+^ mesangial cells (cluster 40) from 0.03% to 0.18%, and CD24^+^CD54^+^K18^+^ collecting duct cells from 0.5% to 13.1%, while remaining tubular epithelial cells were reduced from 95% to 69.5% (**Supplementary Figure 1**).

From 3 days to 15 days post-ligation, we found that the CLR-transformed abundances of all immune cells, except eosinophils, continued to increase along with mesangial cells and CD24^+^CD54^+^K18^+^ collecting duct cells, while other epithelial cells from the ALoH/distal tubules and proximal tubules were further reduced (**Figure 3E**). After 15 days of ligation, CD24^+^CD54^+^K18^+^ collecting duct cells comprised 41% of all cells in the kidney, while the remaining tubular epithelial cells, mesangial cells and immune cells comprised 27%, 2.4% and 20% of all cells, respectively.

### Preventive bemcentinib treatment alleviates fibrosis by reducing abundance of mesangial cells

**Figure 4:**
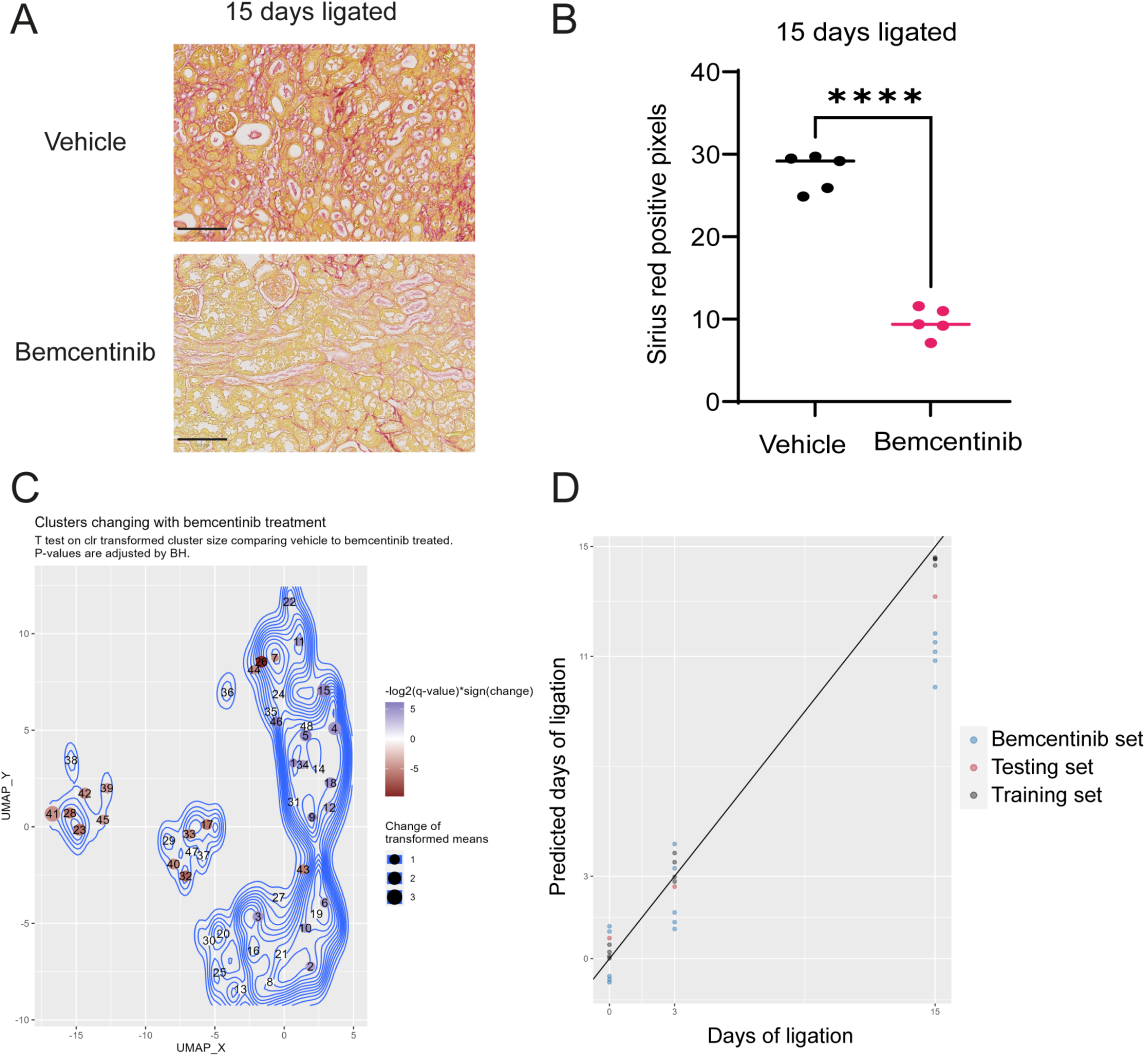
Bemcentinib Alleviates Fibrosis **(A)** Sirius red staining of kidneys ligated for 15 days from mice treated with vehicle or bemcentinib. Scale bar is 100 µm. **(B)** Sirius red quantification. Unpaired two-sample T test with Welch’s correction. ****P < 0.0001. The vehicle group is identical to 15 days ligated vehicle in **Figure 1B**. **(C)** UMAP with clusters colored by significant effect of bemcentinib in kidneys of mice subjected to 15 days ligation. Blue clusters increase, while red clusters decrease, with bemcentinib treatment in kidneys ligated for 15 days. Nonsignificant changes (adjusted P value > 0.05) are white. **(D)** LASSO model estimates days of ligation of kidneys samples from bemcentinib treated mice.

To assess the effect of bemcentinib, we first evaluated sirius red staining intensity (**Figures 4A, B**). These results demonstrated that bemcentinib reduced the level of fibrosis in UUO-operated mice. Upon multivariate testing, we found no differences between bemcentinib treated after 3 days of ligation, however, after 15 days of ligation the treatment groups were significantly different (**Figure 3C**). The difference between the bemcentinib treated and vehicle treated groups were also apparent in the lower left corner of the sample-based UMAP (**Figure 2E**).

Using mass cytometry, we were able to identify specific cell types affected by the treatment. Bemcentinib demonstrated significant effects on multiple cell types on day 15, including a reduction in immune cells, mesangial cells, and CD54^+^ epithelial cells as well as an increase in normal epithelial cells from the collecting duct, ALoH/distal tubules, and proximal tubules (**Figure 4C**). To quantify the effect of bemcentinib, we used our trained LASSO model to predict the number of days post-ligation for the samples from bemcentinib-treated animals. The LASSO model estimated that kidneys treated with bemcentinib and subjected to 15 days of ligation appeared as if they had only been ligated for 11 days, indicating a significant reduction in fibrosis (**Figure 4D**). Similar results were obtained using an alternative partial least squares (PLS) model that was likewise trained on untreated sham and ligated kidneys (**Supplementary Figure 2**).

### AXL expression illuminates therapeutic targets in renal fibrosis

**Figure 5:**
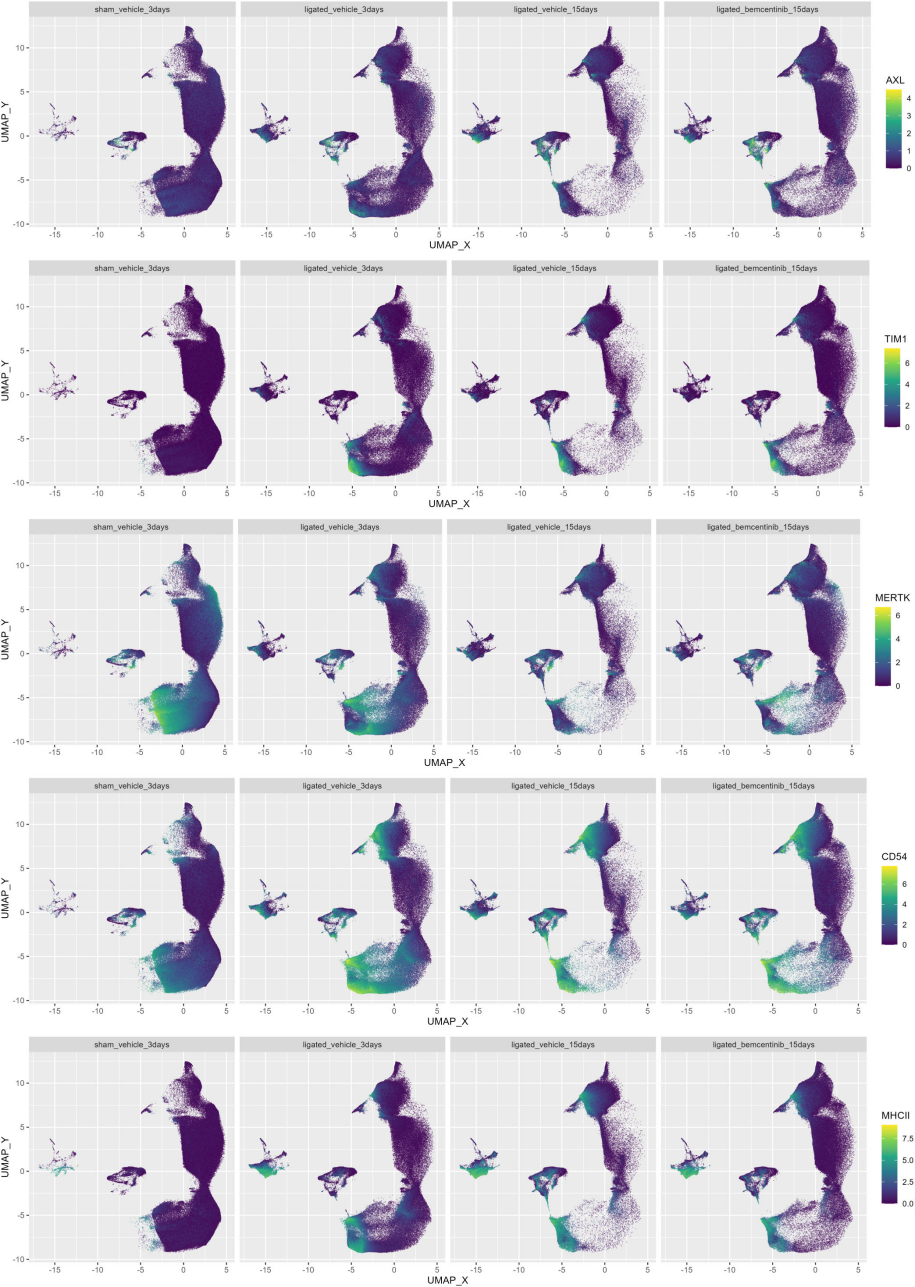
Colored UMAPs highlight relevance of immune and PS-receptors. UMAP of kidneys colored by AXL, CD54, TIM-1, MERTK, MHC-II and signal. Each plot contains the same number of cells.

To evaluate which cell types bemcentinib likely targets to mitigate fibrosis, we measured AXL expression across all clusters. We found that in healthy kidneys, AXL expression was generally very low and mainly found in a subset of endothelial cells (cluster 37) and ASMA^+^ pericytes (cluster 29) (**Figure 5**). At 3 days post-ligation, AXL expression was detectable in multiple additional cell types, including pericytes (cluster 29), an unidentified CD54^+^Vim^+^ cell type (cluster 32), mesangial cells (cluster 40), CD11c^+^ macrophages/DCs (cluster 23) and CD54^+^ proximal tubular epithelial cells (cluster 13, 20, 25, 30). At 15 days post-ligation, AXL was expressed in the same cell types, but at an even higher level. Bemcentinib treatment was also associated with increased mean AXL expression in multiple cell types, such as pericytes (cluster 29), CD54^+^Vim^+^ cells (cluster 32), endothelial cell subset (cluster 37), and mesangial cells (cluster 40). MERTK, another member of the TAM (Tyro3, AXL, MERTK) family of RTKs, which also shares the Gas6 ligand, had a markedly distinct cell type expression pattern compared to AXL. Bemcentinib has previously been demonstrated to display a 50-fold selectivity for AXL compared to MERTK (33). Similar to AXL, we found MERTK expression in endothelial cells and CD163^+^CD206^+^ macrophages, but unlike AXL, MERTK was highly expressed in epithelial cells, particularly from the proximal tubules. In further contrast to AXL, which demonstrated increased staining as fibrosis progressed, MERTK signal diminished during progression. Lastly, as MERTK signal diminished in epithelial cells as fibrosis progressed, the expression of another PS-sensing receptor, TIM1, increased, along with the immune cell adhesion marker CD54 and the antigen presentation complex MHC-II. In summary, we found that AXL expression, initially low in healthy kidneys, increased in specific cell types such as pericytes and mesangial cells with the progression of kidney fibrosis; in contrast, MERTK expression decreased. Treatment with bemcentinib was associated with a further increase in AXL expression in these cells.

## Discussion

Our study utilized mass cytometry to capture temporal cellular changes in the fibrotic kidney including endothelial cells, mesangial cells, epithelial cells, and immune cells. This approach allowed us to discern degrees of fibrosis based on the transformed abundance of these cell types. In general, we found that the progression of fibrosis correlated with a reduction in healthy epithelial cells and an increase in CD54^+^ epithelial cells. Further, mesangial cells and multiple immune cell lineages also increased during fibrosis progression. We leveraged machine learning approaches to determine which cell types were most effective in estimating the degree of fibrotic progression. Despite being based on only three timepoints, the model demonstrated that the transformed abundances of mesangial cells, CD117^+^ Type A intercalated cells, and CD24^+^CD54^+^K18^+^ collecting duct cells were particularly effective in estimating the duration of ligation.

Three days after UUO, we observed a significant increase in mesangial cells and various immune cells including eosinophils, monocytes, and M2 macrophages. However, the most substantial changes were the increase in atypical CD54^+^ epithelial cells in the proximal tubules and collecting duct, and the concurrent loss of epithelial cells covering the segments from proximal tubules to ALoH/distal tubules expressing classical markers. In terms of relative abundance, CD54^+^K18^+^ collecting duct cells increased from 0.5% to 13.1% after 3 days of ligation and to 41% after 15 days of ligation. Considering the initially low abundance of collecting duct tubules observed in sham mice, this increase in collecting duct cells is likely not due to proliferation, but rather phenotypic conversion of existing tubular epithelial cells, possibly from the distal convoluted tubule or ALoH. Previous studies using the UUO model observed increases in K18 expression in collecting ducts, distal tubules and ALoH (34). We show that this increase extends to multiple epithelial lineage markers such as Sca1, EpCAM, and CD24, suggesting these cells acquire traits of collecting duct epithelial cells. Previously, the marker CD24 has been related to injury in tubular epithelial cells (35) and these CD24^+^ cells have been referred to as failed repair tubular epithelial cells (frTEC) (36). The expression of CD54 (ICAM-1) in this population suggests these cells interact with immune cells.

Previous studies have demonstrated that UUO induces an influx of monocytes and macrophages into the kidney (37), importantly they also showed that ablation of monocytes significantly reduced fibrosis, indicating their importance in driving fibrotic progression. We found that UUO led to an increase in monocytes and macrophages along with eosinophils at 3 days post-ligation. On day 15, CD11c^+^ macrophages/DCs and T cells increased while eosinophils decreased. Since eosinophils infiltrate the kidneys early during UUO and later disappear, this suggests that their role diminishes over time, while T cells and DCs which continue to increase likely play a role in the later stages of UUO. While CD8^+^ T cell depletion has been shown to reduce abundance of M2 macrophages along with fibrosis (38), we did not include markers to distinguish between T cell subsets in this study.

We confirmed the previously-reported anti-fibrotic effect of bemcentinib in the UUO model (12), while also identifying the primary AXL-expressing cells in the kidney as pericytes, mesangial cells, and macrophages/DCs, highlighting these cell types in AXL targeted therapy. Some CD54^+^ epithelial cells expressed low levels of AXL, which may represent EMT in epithelial cells during UUO (12). In contrast to AXL-expressing mesangial cells and macrophages/DCs, pericytes, which were also AXL^+^ and uniquely ASMA^+^, did not diminish upon treatment with bemcentinib. Since AXL is a phagocytic receptor, it is possible that selective AXL inhibition with bemcentinib increases phagocytosis through other PS-sensing receptors such as MERTK and TIM1, leading to cellular states important in resolution of fibrosis. As MERTK signal diminished in proximal tubules during fibrotic progression, the expression of another PS-receptor, TIM1, increased. Further, the expression of CD44, CD54, and MHC-II also increased, suggesting an elevated interaction between these proximal epithelial cells and immune cells.

In conclusion, our findings support a mechanistic model wherein bemcentinib treatment alleviates UUO-induced kidney fibrosis by inhibiting mesangial cell proliferation (9) and monocyte-to-macrophage differentiation (39), consistent with the reduction in mesangial cells and macrophages observed in our study. The concomitant reduction in immune infiltration induced by bemcentinib supports the application of AXL-targeting therapeutics to treat inflammation-driven kidney fibrosis.

## Data Availability

The raw data supporting the conclusions of this article will be made available by the authors, without undue reservation.
