# Supplementary material for: Targeting AXL cellular networks in kidney fibrosis
Source: Front Immunol. 2024 Nov 4;15:1446672. doi: 10.3389/fimmu.2024.1446672 (PMC11570270; doi:10.3389/fimmu.2024.1446672)
Supplement: Supplementary file 1 [file DataSheet1.docx]

# Supplementary figures


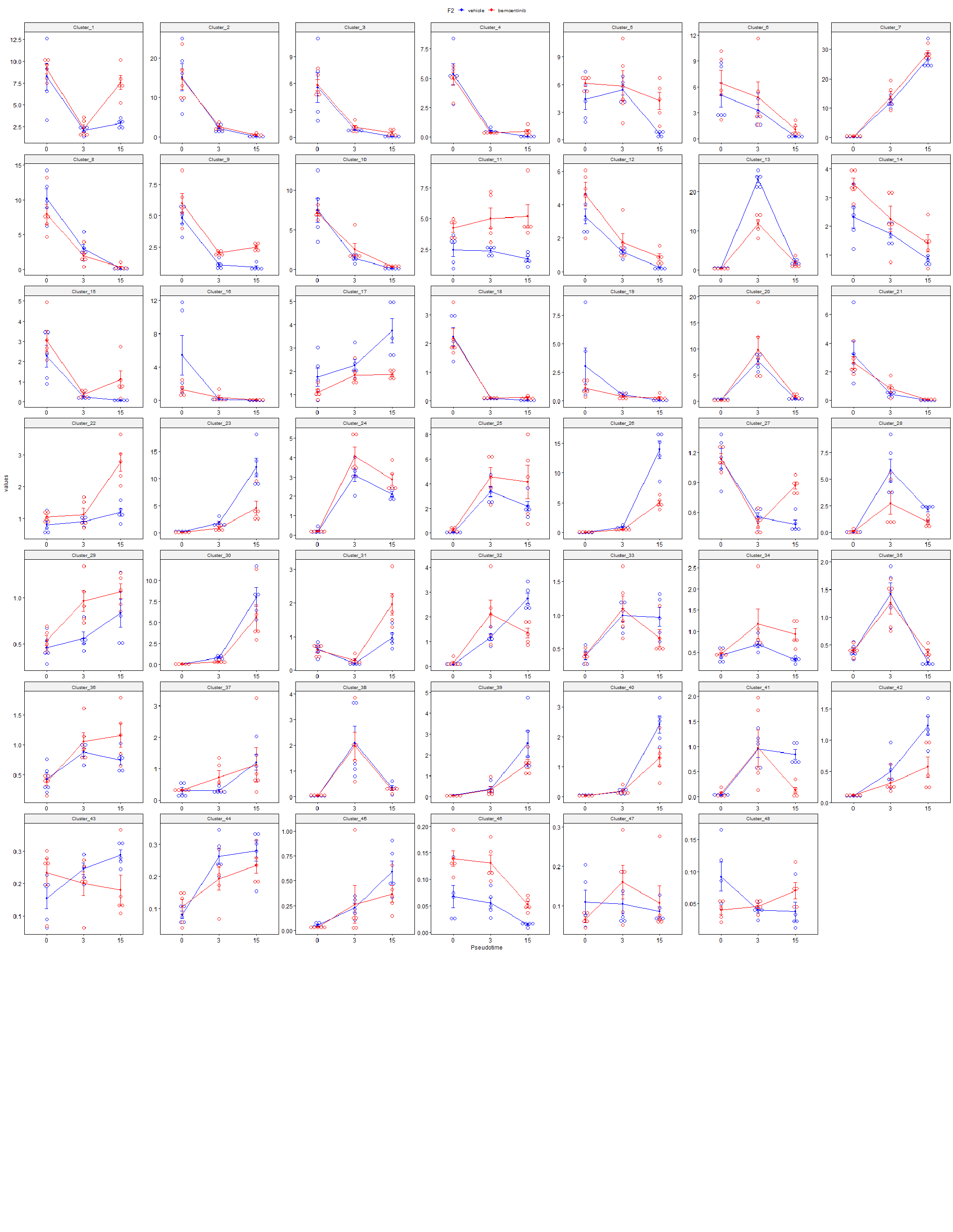


Supplementary Figure 1: **Percentage each cluster makes up in each sample**. Percentage each cluster makes up in each sample across days of ligation and in vehicle (blue) and bemcentinib (red) treatment. Day 0 samples are derived from sham treated kidneys.


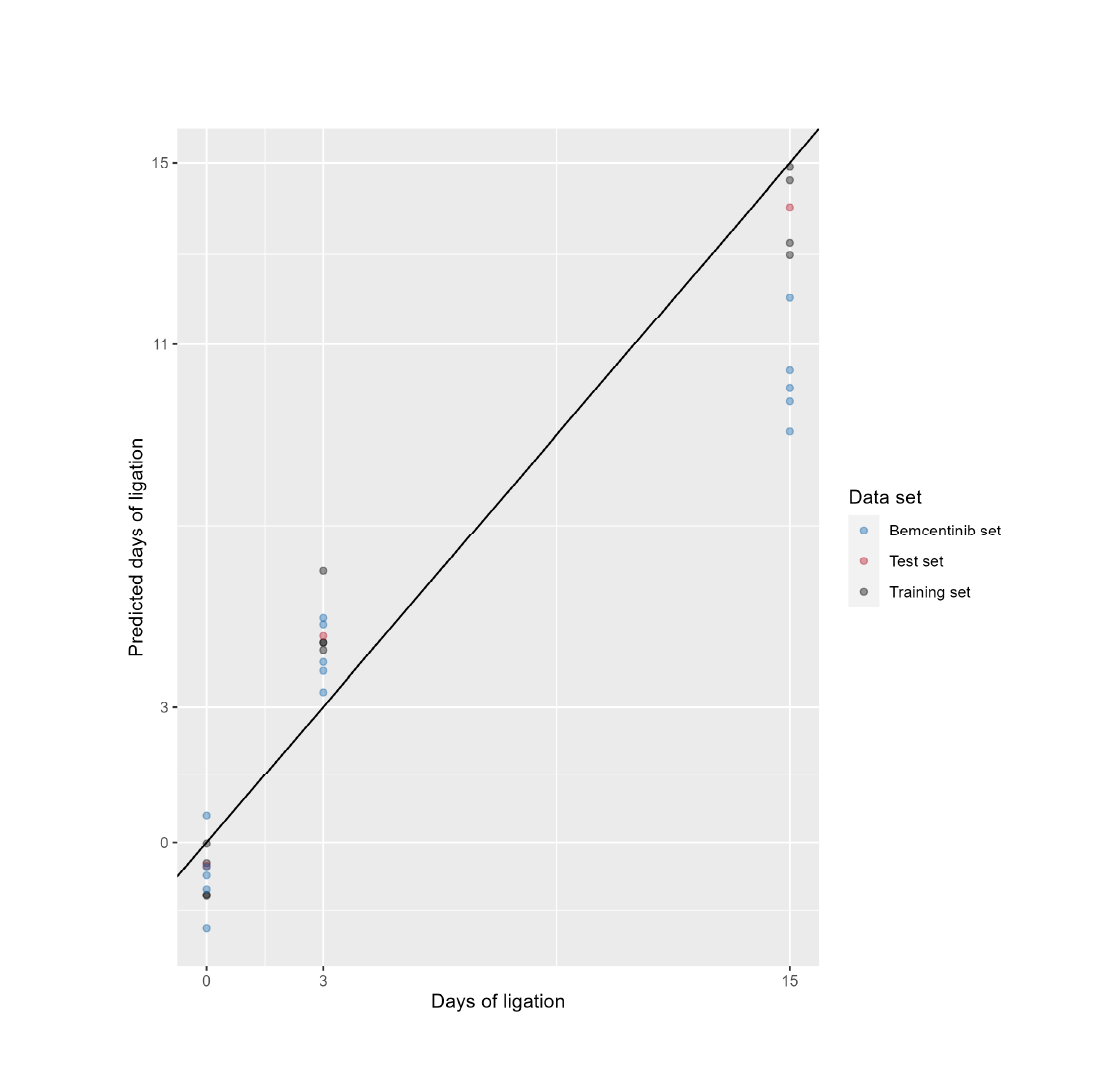


Supplementary Figure 2: **Partial least squares (PLS) regression on vehicle treated samples to predict number of days of ligation based on transformed abundance of each cluster.** The model was then used to predict days of ligation for bemcentinib treated samples.


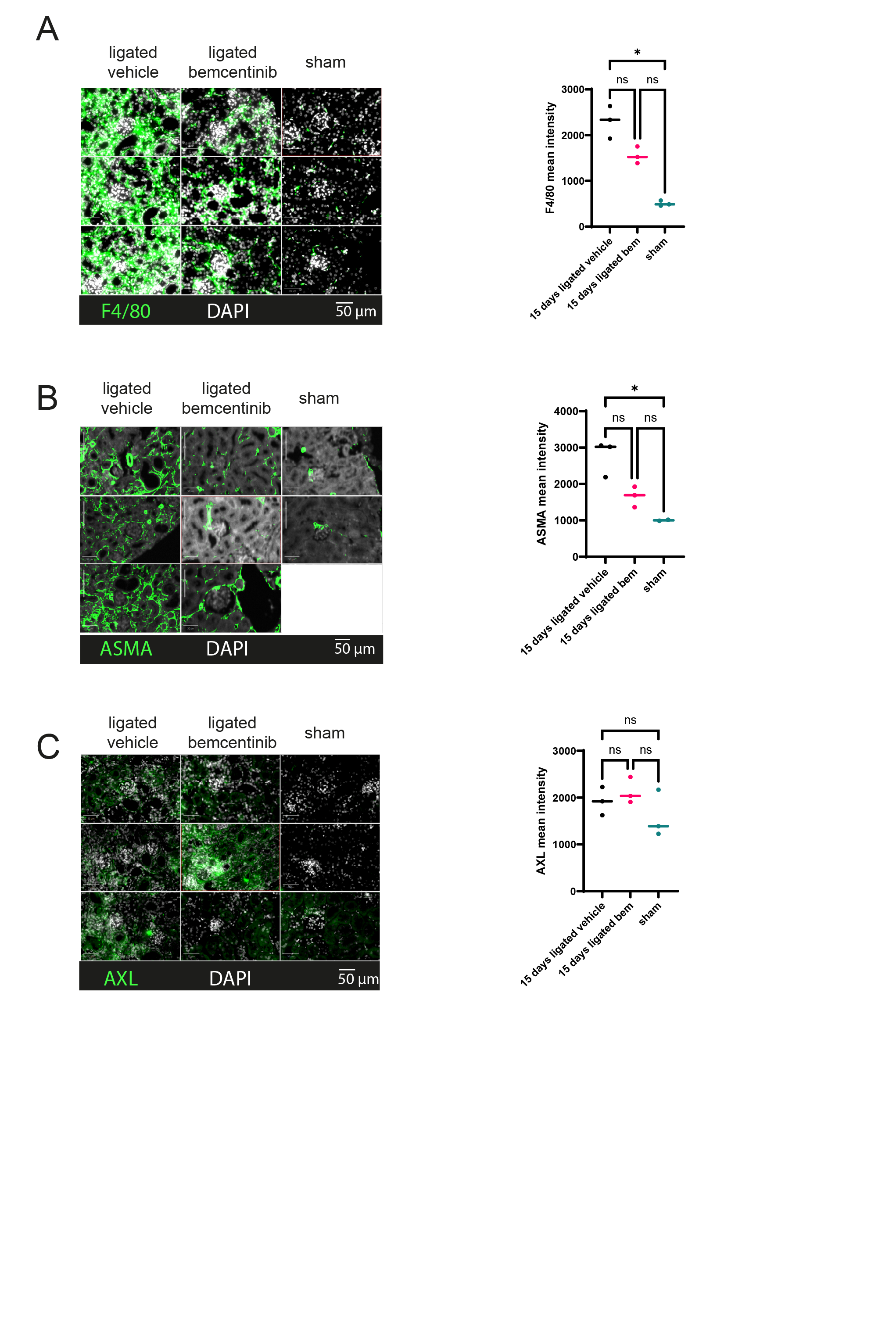


Supplementary Figure 3: **Immunofluorescence imaging of sham mouse kidneys or mouse kidneys ligated and treated with vehicle or bemcentinib for 15 days.** Left panel: Kidneys were stained for F4/80 (**A**), Alpha-smooth muscle actin (ASMA) (**B**), or AXL (**C**). Each sample is zoomed in on the cortex. All images of the same marker have identical color threshold settings. Right panel: Signal intensity of the whole kidney (F4/80 and ASMA), or cortex (AXL) was quantified for each kidney in two or three biological replicates. One sham sample in (**C**) (lower right corner in left panel) demonstrated high background staining of AXL. Kruskal-Wallis (P < 0.05 for F4/80 and ASMA, ns for AXL) followed by Dunn's multiple comparisons test. * P value < 0.05; ns P value > 0.05.

# Supplementary tables

Supplementary table 1: List of antibodies used in mass cytometry. Markers demonstrating nonspecific staining were not used in analysis.

| **Element** | **Mass** | **Target** | **staining location** | **Clone** | **Vendor** | **Cat#** | **Used during analysis** |
| --- | --- | --- | --- | --- | --- | --- | --- |
| Y | 89 | CD45 | Surface | 30-F11 | Fluidigm | 3089005B | Yes |
| Cd | 111 | CD117 | Surface | 2B8 | Thermo Fisher | 14-1171-85 | Yes |
| Cd | 112 | Ly-6A/E | Surface | D7 | Thermo Fisher | 14-5981-82 | Yes |
| Cd | 113 | CD163 | Surface | TNKUPJ | Thermo Fisher | 14-1631-82 | Yes |
| Cd | 114 | TCR gd | Surface | GL3 | Thermo Fisher | 14-5711-82 | Yes |
| In 113/115 | 115 | B220 | Surface | RA3-6B2 | Thermo Fisher | 14-0452-85 | Yes |
| Cd | 116 | TCR beta | Surface | H57-597 | Thermo Fisher | 14-5961-82 | Yes |
| La | 139 | CD34 | Surface | RAM34 | Thermo Fisher | 14-0341-82 | No |
| Ce 140/142 | 140 | TER-119 | Surface | TER-119 | BioLegend | 116202 | No |
| Pr | 141 | Ly-6G | Surface | 1A8 | Fluidigm | 3141008B | Yes |
| Nd | 142 | CD11c | Surface | N418 | Fluidigm | 3142003B | Yes |
| Nd | 143 | ASMA | Intracellular | 1A4 | Thermo Fisher | 14-9760-82 | Yes |
| Nd | 144 | CD326 | Surface | G8.8 | Thermo Fisher | 14-5791-85 | Yes |
| Nd | 145 | CD146 | Surface | ME-9F1 | BioLegend | 134702 | Yes |
| Nd | 146 | CD31 | Surface | 390 | Thermo Fisher | 14-0311-85 | Yes |
| Sm | 147 | Megalin | Surface | CD7D5 | Novus | NB110-96417 | Yes |
| Nd | 148 | THP | Surface | 774056 | R&D | MAB5175 | Yes |
| Sm | 149 | Vimentin | Intracellular | EPR3776 | Abcam | AB193555 | Yes |
| Nd | 150 | CD24 | Surface | M1/69 | Fluidigm | 3150009B | Yes |
| Eu | 151 | CD64 | Surface | X54-5/7.1 | Fluidigm | 3151012B | Yes |
| Sm | 152 | CD324 | Surface | DECMA-1 | Thermo Fisher | 2026-04-30 | No |
| Eu | 153 | PD-L1 | Surface | 10F.9G2 | BioXCell | BE0101 | Yes |
| Sm | 154 | CD11b | Surface | M1/70 | Fluidigm | 3154006B | Yes |
| Gd | 155 | CD90.2 | Surface | 53-2.1 | Thermo Fisher | 14-0902-82 | Yes |
| Gd | 156 | Nestin | Intracellular | Rat-401 | BioLegend | 655102 | No |
| Gd | 157 | CD170 | Surface | 1RNM44N | Thermo Fisher | 14-1702-82 | Yes |
| Gd | 158 | Foxp3 | Intracellular | FJK-16s | Fluidigm | 3158003A | No |
| Tb | 159 | F4/80 | Surface | BM8 | Fluidigm | 3159009B | Yes |
| Dy | 160 | CD44 | Surface | IM7 | Thermo Fisher | 14-0441-82 | Yes |
| Gd | 161 | Desmin | Intracellular | Y66 | Abcam | ab216616 | Yes |
| Dy | 162 | Ly6C | Surface | HK1.4 | Fluidigm | 3162014B | Yes |
| Dy | 163 | CD54 | Surface | YN1/1.7.4 | Fluidigm | 3163020B | Yes |
| Dy | 164 | AQP1 | Surface | EPR11588(B) | Abcam | ab178352 | Yes |
| Ho | 165 | TIM1 | Surface | RMT1-4 | Thermo Fisher | 14-5861-82 | Yes |
| Er | 166 | K18 | Intracellular | RGE53 | Novus | NBP1-97715 | Yes |
| Er | 167 | CD335 | Surface | 29A1.4 | Fluidigm | 3167008B | Yes |
| Gd | 168 | PDGFRb | Intracellular | Y92 | Abcam | AB21597B | No |
| Tm | 169 | CD206 | Intracellular | C068C2 | Fluidigm | 3169021B | Yes |
| Er | 170 | MerTK | Surface | DS5MMER | Thermo Fisher | 14-5751-82 | Yes |
| Er | 170 | MerTK | Surface | 2B10C42 | BioLegend | 151502 | Yes |
| Yb | 171 | CD80 | Surface | 16-10A1 | Fluidigm | 3171008B | Yes |
| Yb | 172 | Caspase3 | Intracellular | 5A1E | Fluidigm | 3172027D | No |
| Yb | 173 | CD71 | Surface | R17217 (RI7 217.1.4) | Thermo Fisher | 14-0711-82 | Yes |
| Yb | 174 | CD18 | Surface | M18/2 | Thermo Fisher | 14-0181-81 | Yes |
| Lu | 175 | Gas6 | Intracellular | Polyclonal | R&D | AF986 | Yes |
| Yb | 176 | Axl | Surface | 107332 | R&D | MAB854 | Yes |
| Yb | 176 | Axl | Surface | 175128 | R&D | MAB8541 | Yes |
| Bi | 209 | I-A/I-E | Surface | M5/114.15.2 | Fluidigm | 3209006B | Yes |
